# Prospective blind comparative clinical study of two point fixation of zygomatic complex fracture using wire and mini plates

**DOI:** 10.1186/1746-160X-8-7

**Published:** 2012-03-06

**Authors:** Lakshmi N Gandi, Vivekanand S Kattimani, Amit V Gupta, V Srinivas Chakravarthi, Sridhar S Meka

**Affiliations:** 1 Department of Oral and Maxillofacial Surgery, Saraswati Dhanwantari Dental College and Hospital, Pathri road, NH222 Parbhani, Maharashtra, India; 2 Department of Oral and Maxillofacial Surgery, Sibar Institute of Dental Sciences, Guntur, AP522509, India; 3 Consultant Maxillofacial Surgeon, Hyderabad, Andhra Pradesh, India

**Keywords:** Zygomatic maxillary complex (ZMC), Internal fixation, Wire osteosynthesis, Mini plate osteosynthesis, Comparison of fixation

## Abstract

**Background:**

The zygomatic maxillary complex (ZMC) fractures are one of the most frequent injuries of the facial skeleton due to its position and facial contour. Assaults, road traffic accidents and falls are the principal etiologic factors that may cause fractures of zygomatic bone. The different fixation methods are applied to treat the zygomatic bone fractures, with many more classifications which have been described in the literature for the ease of management. The type of the fracture, its severity and associated facial fractures usually interferes the treatment modality.

**Purpose of study:**

The aim of this paper is to show the results of 18yrs prospective blind comparative study using wire and plate osteosynthesis which needed open reduction and internal fixation involving Type II to Type IV Spissel and Schroll ZMC fractures.

**Materials and methods:**

Total 80 cases included in the study out of 1780 ZMC cases which were treated using wire and plate osteosynthesis over a period of 18 yrs, involving only Type II to Type IV Spissel and Schroll ZMC fractures. Other types excluded from study to prevent observer bias. All the fixations carried out through Standard Dingman's incision using stainless steel 26 gauze wire and titanium 1.5 mm mini plate system under general anesthesia by single maxillofacial surgeon and evaluated by another maxillofacial surgeon who is blinded for surgical procedure after 2 and 4 wks of follow-up for facial symmetry, wound healing, functional assessment (mouth opening, diplopia), and sensory disturbance. All the data tabulated in Excel software (Microsoft) for statistical analysis. P-value calculated to know the Significance of treatment modality in all aspects.

**Results:**

Result shows no significant p-values indicating both the operating techniques are equally efficient in the surgical management of ZMC fracture.

**Conclusion:**

Osteosynthesis by mini plates is simple, logical and effective treatment compared to wire osteosynthesis in regard to stability of fracture fragments. Wire osteosynthesis will be helpful in emergency surgeries or where the mini plates are not available. Even though the wire osteosynthesis is economical compared to mini plate fixation; but the time and skill is required for fixation of wires.

## Introduction

The zygomatic complex fractures are one of the most frequent injuries to the facial skeleton due to its position and facial contour [1]. It is quadrilateral bone with irregular convex external surface and concave internal surface having four processes, which articulates with the frontal, maxillary, temporal and sphenoid bones. Its external surface forms the prominence of the cheek. The zygomatic complex has robust joint with the maxilla and weak linking's with the sphenoid and the narrow zygomatic process of the temporal bone [1].

Some classifications for the zygomatic bone fractures were described in the literature [2-6]. Trauma of the zygomatic complex constitutes about 45% of the fractures of middle third of the face [7]. Etiology cites the physical aggressions, falls and road traffic accidents [7,8]. The prevalence age for the fractures of the zygomatic bone varies from 21 to 40 years [8].

Many surgical treatments have been proposed for reduction of the zygomatic bone fractures, in accordance with the severity of the fracture, its extension, and other associated fractures and orbital floor involvement [1]. The steel wire use is cited in the literature beyond the internal fixation with mini plates and screws [9,10].

With the advent of the Internal Fixation, the surgical treatment started to provide greater previsibility of results for the reduction of the fractures of the zygomatic bone, as well as assists in the postoperative rehabilitation for more satisfactory form, function and esthetics with less comorbidity.

## Aim and objectives

Purpose of the study is to prospectively compare the two point fixation of zygomatic complex fracture using wire and mini plates for facial symmetry, wound healing, functional assessment (mouth opening, diplopia), and sensory disturbance.

## Materials and methods

Study group consisted total of 80 Cases out of 1780 ZMC cases which were treated using wire and plate osteosynthesis over a period of 18 yrs with Zygomatic complex fracture classified according to Spissel and Schroll [6], as Type I -Isolated Zygomatic arch fracture, Type II-Fracture with no significant displacement, Type III-partially displaced medially, Type IV-Totally displaced medially, Type V-Those with dorsal displacement, Type VI-Those with inferior displacement, Type VII-comminuted fractures. In our study we considered only Type II to Type IV fractures, grouped in to two major categories depending on the type of fixation as wire (Category A- 40 cases) and mini plate (Category B- 40 cases) and subdivided in to 3 groups as Type II (25%), Type III (37.5%), Type IV (37.5%) in each; Which Consisting of type III and IV ZMC fractures 15 cases each and Type II fractures 10 cases each. (Refer Table 1: Distribution of cases)

**Table 1:** Distribution of cases according to Spissel and Schroll

Category	Type II	Type III	Type IV	Total No. of cases
A (fixation with wire)	10	15	15	40

B (fixation with mini plate)	10	15	15	40

Total No. of cases	20	30	30	80

### Surgical method

Surgical intervention carried out in all ZMC fractures where 1) Radiographic evidence of displacement, 2) A palpable step or discrepancy in the orbital rim or zygomatic arch 3) Exophthalmos and extra ocular muscle dysfunction found. After assessing the general condition of patient, all cases were treated randomly under general anesthesia. The standard Dingmans's Incision used in all cases to expose and fix the fracture fragments. All patients put on prophylactic antibiotics for 5 days post operatively. Suture removed after 8 days post operatively. For transosseous wiring we used 26 gauze stainless steel pre stretched wire; which twisted to approximate the fractured fragments after reduction and stabilization. The titanium 1.5 mm two holed plate and 4 holed orbital plates fixed with 1.5 × 6 mm screws.

### Clinical observations and Results

**Figure 1:**
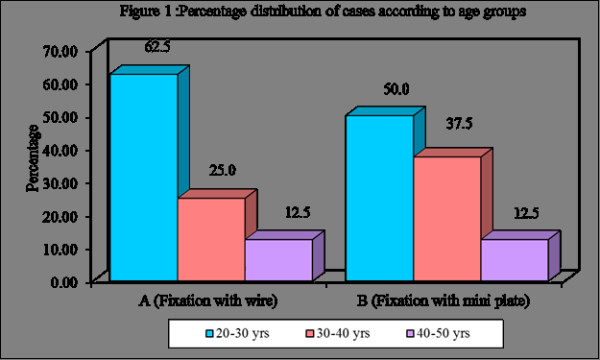
**Graph showing Age distribution among the treated cases**.

**Figure 2:**
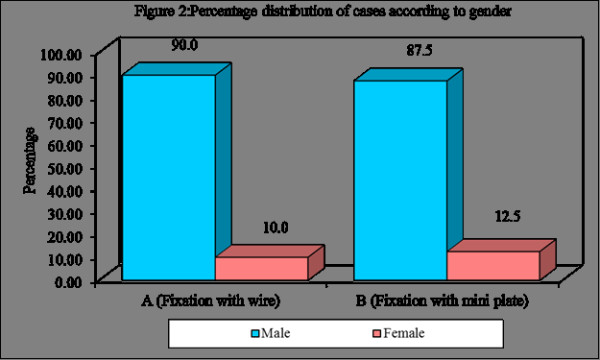
**Graph showing Sex distribution among the treated cases**.

Figure 1, Figure 2, Table 2, Table 3, Table 4

**Table 2:** Pre operative evaluation of Parameters among the treated groups

Parameters/Category	Type II	Type III	Type IV
Facial asymmetry	15	30	30

Occlusion disturbance	0	0	0

Restricted Mouth opening	3	30	30

Sensory disturbance	2	30	30

Diplopia	0	18	20

**Table 3:** Post operative evaluation of Parameters among the treated groups

Category 1	Parameters/groups	Type II	Type III	Type IV
Wire osteosynthesis 40 Patients	Facial asymmetry	Normal	Normal	1
	
	Occlusion disturbance	No	No	No
	
	Restricted Mouth opening	No	No	No
	
	Sensory disturbance	No	No	1 recovered after 3 months
	
	Diplopia	absent	absent	absent

**Table 4:** Post operative evaluation of Parameters among the treated groups

Category 2	Parameters/groups	Type II	Type III	Type IV
Mini plate osteosynthesis 40 Patients	Facial asymmetry	Normal	Normal	No
	
	Occlusion disturbance	No	No	No
	
	Restricted Mouth opening	No	No	No
	
	Sensory disturbance	No	No	2 recovered after 3 months
	
	Diplopia	absent	absent	absent

Among the 80 cases treated 71 were males and 9 were females. Age distributions of the patients were 20-50 yrs. Among cases treated one case in wire osteosynthesis showed mild facial asymmetry due to preexisting tissue loss, which was of more soft tissue defect. This problem of scar contracture was planned to be corrected by plastic surgical procedure.

All cases were treated randomly by maxillofacial surgeon, and evaluated by another maxillofacial surgeon who was blinded for the surgical procedure involved. All the results were tabulated and analyzed with statistical analysis to find significance between the both types of treatment. Both the operating techniques are equally efficient in the surgical management of ZMC fracture as results show no significant P values.

## Discussion

The ZMC is a functional and aesthetic unit of the facial skeleton. This complex serves as a bony barrier, separating the orbital constituents from the maxillary sinus and temporal fossa. The zygoma has 4 bony attachments to the skull, and ZMC fractures are sometimes known as tetrapod fractures [2-7]. Trauma to the ZMC can result in multiple fractures, but solitary bony disruption may occur, as with isolated zygomatic arch fracture. The zygoma is the second most commonly fractured facial bone [6,7]. Deformity of face consists of depression or flattening of cheek bone [6].

Today fractures of ZMC are receiving increased attention because of increase in incidence and recognition of direct involvement with the contents of orbital cavity, particularly the extra ocular muscles and periorbital tissue [11]. Since mid-face injuries heal rapidly because of the nature of bone and consolidate earlier than other bones. Early intervention is necessary to achieve cosmetic and functional results. Various methods for the repair of ZMC have been advocated with emphasis on different methods of fixation. The aim of any treatment is to give best results with least morbidity [12].

One of the popular modalities of treatment involves the reduction of fractured fragments through Dingman's approach. Any mobility of fracture fragments impedes healing. Therefore in addition to accurate reduction, fixation is often necessary to achieve healing of fractured bone [13]. Reduction and fixation of fracture of ZMC by Dingaman's incision offers number of advantages according to Zingmunt W.Pozatek [13]. In our study reduction and fixation done by transosseous wiring or mini plates using Dingaman's incision. Our findings agree with Zingmunt W.Pozatek [13] regards to scar which confines within eyebrow and it is imperceptible.

Open reduction and transosseous wiring done in 40 cases in relation to frontozygomatic suture and zygomatico maxillary suture which were held in anatomical position. Our study correlates with the study conducted by Pozatek et al who used transosseous wiring at frontozygomatic suture [13]. Rest of the wiring was done at zygomatico maxillary suture which is in accordance with study conducted by Altonen et al [14]. Manson et al. [7] But the twisting the ligatures generates uncontrollable pressure which leads to tearing of suture in bones.

But because of mini plate osteosynthesis the all older methods of fixation techniques have not used regularly. The miniplate fixation resulted in ten times higher strength than wire osteosynthesis [15]. This is in concurrence with the results obtained in our study.

In the present study no displacement of zygoma occurred after fixation. But in one case little asymmetry existed due to soft tissue loss that resulted in esthetic compromise which correlates with findings of Jackson, Kunio Ikemura and Keith [15-17]. With regard to ophthalmic signs like diplopia, our results obtained were in accordance with the results of Keith et al [17]. Three neurological deficits were found in our study for 2-3 months and resolved gradually after 3 months. Both study showed no statistical difference in there inference but the clinical experience reveals the miniplate fixation gives more ease for the surgeon than wire osteosynthesis. Sometime it's difficult to prevent medial rotation of fractured arch fragment with only two point fixation using wires; but it is not so in mini plate osteosynthesis. Even though the wire osteosynthesis is economical compared to mini plate fixation; but the time and skill is required for fixation of wires.

## Conclusion

Osteosynthesis by mini plates is a simple, logical and effective treatment compared to wire osteosynthesis as regard to stability of fracture fragments. Currently this is most accepted and followed method in our center. Wire osteosynthesis will be helpful in emergency surgeries, or where the availability of mini plates is not possible.

## Competing interests

The authors declare that they have no competing interests.

## Authors' contributions

LNG started the study with help of VSC. LNG and VSK carried out the drafting of article, SSM, AVG and VSC carried out the observations and tabulation subsequently. The revision and results with conclusion drafted by VSK and AVG. The overall revision and discussion has been drafted by VSK, LNG and SSM. Revised by VSK and VSC. All authors read and approved the final manuscript.
